# Contemporary Data on Sex Differences in Coronary Angiography Findings: A Dual-Nation Study

**DOI:** 10.1016/j.jacadv.2026.102807

**Published:** 2026-06-17

**Authors:** Mikael Zhou, Sacharias von Koch, Kevin Kris Warnakula Olesen, Pernille Gro Thrane, Michael Maeng, Sasha Koul, Karolina Szummer, Giovanna Sarno, Axel Dahlgren, Eva Swahn, Tomas Jernberg, Moman A. Mohammad, David Erlinge

**Affiliations:** a Department of Cardiology, Clinical Sciences, Lund University, Skane University Hospital, Lund, Sweden; b Department of Cardiology, Aarhus University Hospital, Aarhus, Denmark; c Department of Clinical Sciences, Danderyd Hospital, Karolinska Institute, Stockholm, Sweden; d Department of Medical Sciences, Cardiology, Uppsala Universitet, Uppsala, Sweden; e Department of Cardiology, and Department of Health, Medicine and Caring Sciences, Linköping University, Linköping, Sweden

**Keywords:** coronary artery disease, coronary angiography, NSTE-ACS, SCAAR, sex differences, Western Denmark heart registry

## Abstract

**Background:**

Sex differences in the diagnostic yield of invasive coronary angiography (ICA) in non–ST-elevation acute coronary syndrome (NSTE-ACS) are well established, but contemporary population-level data incorporating high-sensitivity troponin era cohorts and cross-national validation remain limited.

**Objectives:**

The objectives of the study were to assess sex differences in diagnostic yield, revascularization rates, and 1-year outcomes in patients undergoing first-time ICA for suspected NSTE-ACS.

**Methods:**

Using data from the Swedish Coronary Angiography and Angioplasty Registry with validation in the Western Denmark Heart Registry. Adults without previously known coronary artery disease (CAD) undergoing first-time ICA for suspected NSTE-ACS were included. The primary outcome was the absence of significant CAD, defined as a normal angiography or <50% stenosis in all major epicardial vessels. Multivariable Poisson regression models were used to estimate adjusted risk ratios (aRRs). Secondary outcomes were 1-year major adverse cardiovascular events, comparing women and men with and without obstructive CAD using Kaplan-Meier estimates and adjusted Cox proportional hazards models.

**Results:**

A total of 74,883 and 17,863 patients were included from Sweden and Western Denmark. Women had approximately twice the proportion without significant CAD compared to men in Swedish Coronary Angiography and Angioplasty Registry (40.9% vs 17.2%; aRR: 2.59; 95% CI: 2.51-2.67) and Western Denmark Heart Registry (50.1% vs 25.2%; aRR: 2.01; 95% CI: 1.95-2.08). In women below the age of 50 without any traditional cardiovascular risk factors, 74.8% had no significant CAD.

**Conclusions:**

In 2 population-based cohorts undergoing first-time ICA for suspected NSTE-ACS, 40 to 50% of women had no significant CAD, approximately twice the proportion to men, highlighting ongoing challenges in risk stratification of patients referred for ICA.

Patients with ST-segment elevation myocardial infarction (MI) require immediate invasive coronary angiography (ICA), whereas patients with suspected chronic coronary syndrome, or a low-to-moderate likelihood of obstructive coronary artery disease (CAD), are preferentially referred for noninvasive diagnostics.^1^^,^^2^ For patients presenting with non–ST-segment elevation acute coronary syndrome (NSTE-ACS), the timing and choice of diagnostic modality are guided by clinical risk stratification. Current American Heart Association and European Society of Cardiology guidelines recommend a routine invasive approach for patients with unstable, high-risk, or intermediate-risk features, with timing ranging from immediate to within 72 hours, depending on risk category.^1^^,^^3^ In patients with low-risk features, either noninvasive risk stratification or ICA before discharge is considered appropriate. These recommendations are informed by meta-analyses of randomized controlled trials, as well as the VERDICT and TIMACS trials, which showed no clear long-term advantage of an early invasive approach in terms of major adverse cardiovascular events (MACE) in patients presenting with NSTE-ACS.^4^, ^5^, ^6^ However, among the highest risk patients, defined as GRACE score >140, an early approach was associated with improved outcomes.

The adoption of high-sensitivity troponin assays has enhanced the rapid rule out of MI but has simultaneously increased the detection of minor myocardial injury, creating a diagnostic challenge in patient selection for ICA.^7^ ICA remains the reference standard for diagnosing obstructive CAD, and allows for ad hoc percutaneous coronary intervention (PCI). However, ICA carries low but clinically relevant risks; therefore, appropriate patient selection is essential to minimize procedural complications and optimize resource utilization. Prior studies investigating the rate of nonobstructive CAD at ICA in an NSTE-ACS cohorts have shown a prevalence ranging between 12 to 37% among women and 9 to 16% among men. Most of these studies are based on older cohorts, preceding the widespread adoption of high-sensitivity troponins.^8^, ^9^, ^10^, ^11^, ^12^, ^13^, ^14^, ^15^, ^16^ In addition, there is a lack of contemporary data on subsequent revascularization patterns and clinical outcomes by sex in NSTE-ACS patients.

Despite advances in risk stratification, sex-based differences in the diagnostic yield of ICA persist, with women consistently more likely than men to have nonobstructive CAD at angiography. Therefore, the present study aimed to evaluate the contemporary diagnostic yield of ICA in a large, unselected cohort undergoing first-time ICA for suspected NSTE-ACS, with a specific focus on identifying sex differences and subsequent revascularization patterns and clinical outcomes.

## Methods

### Data sources

Data were obtained from The Swedish Coronary Angiography and Angioplasty Registry (SCAAR) and Western Denmark Heart Registry (WDHR). SCAAR prospectively collects data from all catheterization laboratories in Sweden, including patient characteristics, segment characteristics, and procedural information.^17^ Data from SCAAR were linked to the National Patient Registry (inpatient diagnoses, according to the International Classification of Disease-10 [ICD-10]), and the National Population Registry (vital status). The Swedish National Patient Registry has been externally validated, with reported diagnostic accuracies of 85 to 95% for cardiovascular diagnoses.^18^

WDHR prospectively collects data from all cardiac interventions performed in Western Denmark, covering approximately 54% of the total Danish population.^19^^,^^20^ Linkage to other registries is made possible through a unique personal identification number, allowing linkage to the Civil Personal Registry (vital status), National Patient Registry, and the Register of Cause of Death. An external review of the Danish National Patient Registry on cardiovascular diagnoses showed a mean positive predictive value of 88% for cardiovascular diagnoses.^16^

The SCAAR study was approved by the Swedish Ethical Review Authority (Dnr 2023-00201-01). The WDHR study was approved by a regional branch of the Danish Data Protection Agency (record no. 1-16-02-193-18). On approval by the regional data protection authorities (record no. 14-45-70-24-22), patient consent requirements were waived.

### Study design, study population, and outcomes

**Figure 1:**
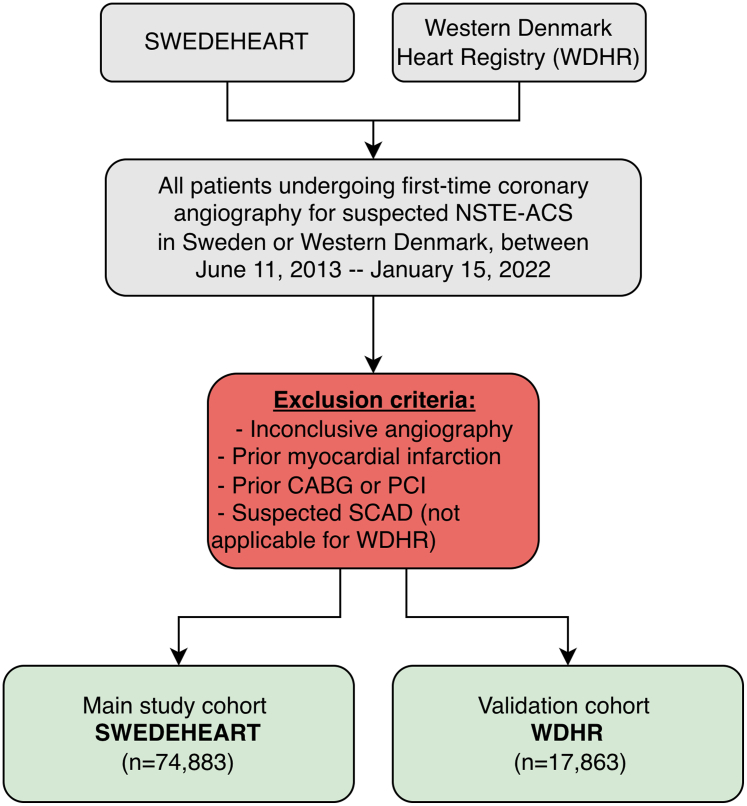
**Inclusion and Exclusion Criteria of Study Population** CABG = coronary artery bypass grafting; NSTE-ACS = non–ST-segment elevation acute coronary syndrome; PCI = percutaneous coronary intervention; SCAD = spontaneous coronary artery dissection.

This was a nationwide observational study, with validation in a comparable cohort from Western Denmark. Inclusion and exclusion criteria are presented in Figure 1.

All adults (age ≥18 years) undergoing a first-time ICA for clinically suspected NSTE-ACS between June 11, 2013, and January 15, 2022, were eligible. Patients with known obstructive CAD, defined as a history of prior MI, coronary artery bypass grafting, or PCI, were excluded (n = 34,158 [29.7%]), as were patients with inconclusive angiographic findings (n = 97 [0.08%]) or suspected spontaneous coronary artery dissection (SCAD) (n = 410 [0.36%]). The same inclusion and exclusion criteria were applied to the Danish cohort (patients with prior CAD, n = 1,747 [8.9%]). SCAD is not recorded in the WDHR. The extent of CAD was reported by the performing interventional physician, and procedural characteristics and in-laboratory complications were recorded prospectively.

The primary objective was to describe the prevalence of nonobstructive CAD in patients with suspected NSTE-ACS undergoing first-time ICA, and to assess sex differences in this outcome. Secondary objectives were to compare revascularization rates between men and women with obstructive CAD, and to evaluate 1-year risks of MACE and all-cause death in men and women with and without obstructive CAD.

The main outcome was the angiographic extent of CAD at ICA, classified as significant (≥50% stenosis) or no significant CAD (normal angiogram or <50% stenosis). Obstructive CAD was defined as at least 1 luminal stenosis occluding ≥50% of the luminal diameter in any major epicardial vessel. Secondary outcomes were: 1) 1-year incidence of MACE, defined as a composite of all-cause death, incident MI, and ischemic stroke; and 2) revascularization, defined as PCI or coronary artery bypass grafting within 30 days of index ICA. Incident MIs recorded within 30 days of index angiography were not included because their validity is considered to be low. Incident MI was defined as a registered hospitalization with MI (ICD-10 code I21). Ischemic stroke was defined as a registered hospitalization with ischemic stroke (ICD-10 code I63) in the Swedish cohort, and as ischemic or unspecified stroke (ICD-10 codes I63–I64) in the Danish cohort.

### Statistical analysis

Continuous data are presented as the median with 25th-75th percentiles (Q1-Q3), and comparisons between groups were performed using Mann-Whitney U test. Categorical variables are presented as counts and percentages, and comparisons were calculated using the chi-square test.

For the primary analysis, unadjusted and adjusted Poisson regression models were used to evaluate relative sex differences in angiographic findings with results presented as the adjusted risk ratio (aRR) with 95% CI. Adjusted models included prespecified demographic and clinical covariates. The same covariates were used in adjusted analyses of revascularization rates.

One-year MACE rates were estimated using Kaplan-Meier curves. Groups were compared using the log-rank test, whereas rates are presented using cumulative incidence percentages. Groups were also compared using multivariable Cox regression models with results presented as the adjusted HR (aHR) with 95% CI. Separate models were constructed for patients with and without obstructive CAD. Full details of covariates included in each model are provided in the corresponding table footnote or figure legend.

To illustrate the proportion of patients without significant CAD across age, sex, and cardiovascular risk factor profiles, a heat map was constructed stratified by sex, age groups, and combinations of traditional cardiovascular risk factors.

All analyses were conducted on a complete case basis due to low levels of missing data (<10%). Statistical analyses were performed using Stata/SE (version 19.5; StataCorp LLC), and the heat map was generated using R (version 4.5.1; R Core Team, 2025).

## Results

### Study population and angiography findings – SCAAR and WDHR

A total of 74,883 patients (35.5% women) in Sweden underwent a first-time ICA for suspected NSTE-ACS. Women were, on average, 4 years older than men at first-time ICA and had a higher comorbidity load (Table 1). In the Swedish cohort, the median age (Q1–Q3) was 63 to 68 years in men and 69 to 72 years in women, depending on CAD status.

**Table 1:** Baseline Characteristics – Swedish Patient Cohort

	Men		Women	
	OCAD (n = 8,321)	NOCAD (n = 39,962)	OCAD (n = 10,883)	NOCAD (n = 15,717)
Age, years; median (Q1-Q3)	63 (54-72)	68 (59-75)	69 (60-76)	72 (65-79)
Inclusion period				
2013-2017	3,987 (47.9%)	21,545 (53.9%)	5,428 (49.9%)	8,556 (54.4%)
2018-2022	4,334 (52.1%)	18,417 (46.1%)	5,455 (50.1%)	7,161 (45.6%)
BMI category				
Underweight	62 (0.7%)	149 (0.4%)	284 (2.6%)	255 (1.6%)
Normal	2,257 (27.1%)	10,916 (27.3%)	4,002 (36.8%)	5,187 (33.0%)
Overweight	3,325 (40.0%)	18,286 (45.8%)	3,485 (32.0%)	5,615 (35.7%)
Obese	2,677 (32.2%)	10,611 (26.6%)	3,112 (28.6%)	4,660 (29.6%)
Diabetes mellitus	1,380 (16.6%)	8,715 (21.8%)	1,484 (13.6%)	3,888 (24.7%)
Hypertension	4,221 (50.7%)	22,315 (55.8%)	6,296 (57.9%)	10,647 (67.7%)
Hyperlipidemia	2,391 (28.7%)	12,392 (31.0%)	3,085 (28.3%)	5,370 (34.2%)
Previous stroke	419 (5.0%)	2,215 (5.5%)	511 (4.7%)	976 (6.2%)
Congestive heart failure	257 (3.1%)	803 (2.0%)	319 (2.9%)	547 (3.5%)
Chronic kidney failure	717 (8.6%)	5,237 (13.1%)	2,396 (22.0%)	4,907 (31.2%)
Peripheral artery disease	167 (2.0%)	1,305 (3.3%)	187 (1.7%)	660 (4.2%)
Urgency				
Elective	810 (9.7%)	3,854 (9.6%)	1,103 (10.1%)	1,520 (9.7%)
Subacute	6,332 (76.1%)	29,289 (73.3%)	8,250 (75.8%)	11,500 (73.2%)
Acute	839 (10.1%)	5,000 (12.5%)	1,111 (10.2%)	2,003 (12.7%)
Missing	340 (4.1%)	1,819 (4.6%)	419 (3.9%)	694 (4.4%)
Ever smoker	1,272 (15.7%)	6,911 (17.8%)	1,422 (13.5%)	3,053 (20.1%)
Angiography indication				
Unstable angina	4,207 (50.6%)	11,835 (29.6%)	3,968 (36.5%)	4,211 (26.8%)
NSTEMI	4,114 (49.4%)	28,127 (70.4%)	6,915 (63.5%)	11,506 (73.2%)
Number of cardiovascular risk factors				
0	2,515 (30.2%)	10,248 (25.6%)	2,883 (26.5%)	3,201 (20.4%)
1	2,633 (31.6%)	12,280 (30.7%)	3,842 (35.3%)	4,978 (31.7%)
2	1,823 (21.9%)	9,690 (24.2%)	2,728 (25.1%)	4,412 (28.1%)
3	1,041 (12.5%)	5,918 (14.8%)	1,203 (11.1%)	2,548 (16.2%)
4	309 (3.7%)	1,826 (4.6%)	227 (2.1%)	578 (3.7%)
Angiographic findings				
Normal/atheromatosis	8,321 (100.0%)	0 (0.0%)	10,883 (100.0%)	0 (0.0%)
1-vessel disease	0 (0.0%)	17,277 (43.2%)	0 (0.0%)	8,040 (51.2%)
2-vessel disease	0 (0.0%)	10,723 (26.8%)	0 (0.0%)	3,833 (24.4%)
3-vessel disease or LM	0 (0.0%)	11,962 (29.9%)	0 (0.0%)	3,844 (24.5%)

LM = left main; NOCAD = nonobstructive coronary artery disease; NSTEMI = non–ST-segment elevation myocardial infarction; OCAD = obstructive coronary artery disease; Q1–Q3 = 25th–75th percentiles.

BMI categories (kg/m^2^): Underweight, BMI <18.5; normal weight, BMI 18.5-24.9; overweight, BMI 25.0-29.9; obese, BMI ≥30.

In Denmark, 17,863 patients (38.8% women) underwent a first-time ICA for suspected NSTE-ACS during the same study period. The median age (Q1–Q3) ranged from 62 (52-71) to 66 (57-74) in men and from 67 (57-75) to 71 (61-79) in women. Detailed baseline characteristics stratified by sex and CAD status are presented in Supplemental Table 2.

### Sex differences in angiography findings

**Figure 2:**
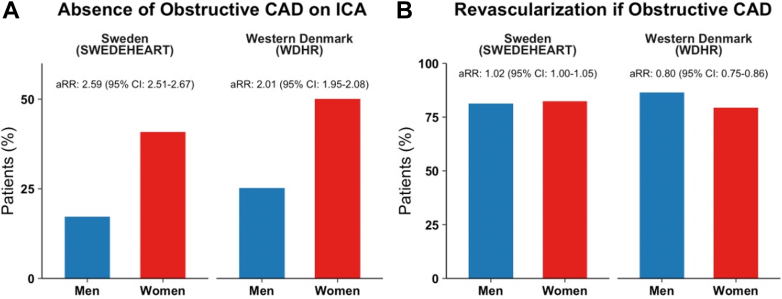
**Sex Differences: Angiographic Findings and Subsequent Revascularization in 2 Cohorts** Prevalence of nonobstructive coronary artery disease (CAD) and rates of subsequent revascularization in patients with significant CAD among patients undergoing invasive coronary angiography (ICA) for clinical indication of suspected non–ST-segment elevation acute coronary syndrome (NSTE-ACS), stratified by sex. (A) Shows the prevalence of patients found to have nonobstructive CAD in Sweden and Denmark. (B) Shows the subsequent rate of revascularization among patients found to have obstructive CAD in the same cohorts. Adjusted rate ratios (aRR) with 95% CIs are shown for women with men as the reference group. Both models were adjusted for age, body mass index, diabetes mellitus, hypertension, hyperlipidemia, chronic kidney disease, congestive heart failure, smoking status, and indication for ICA (NSTEMI or unstable angina). WDHR, Western Denmark Heart Registry.

**Figure 3:**
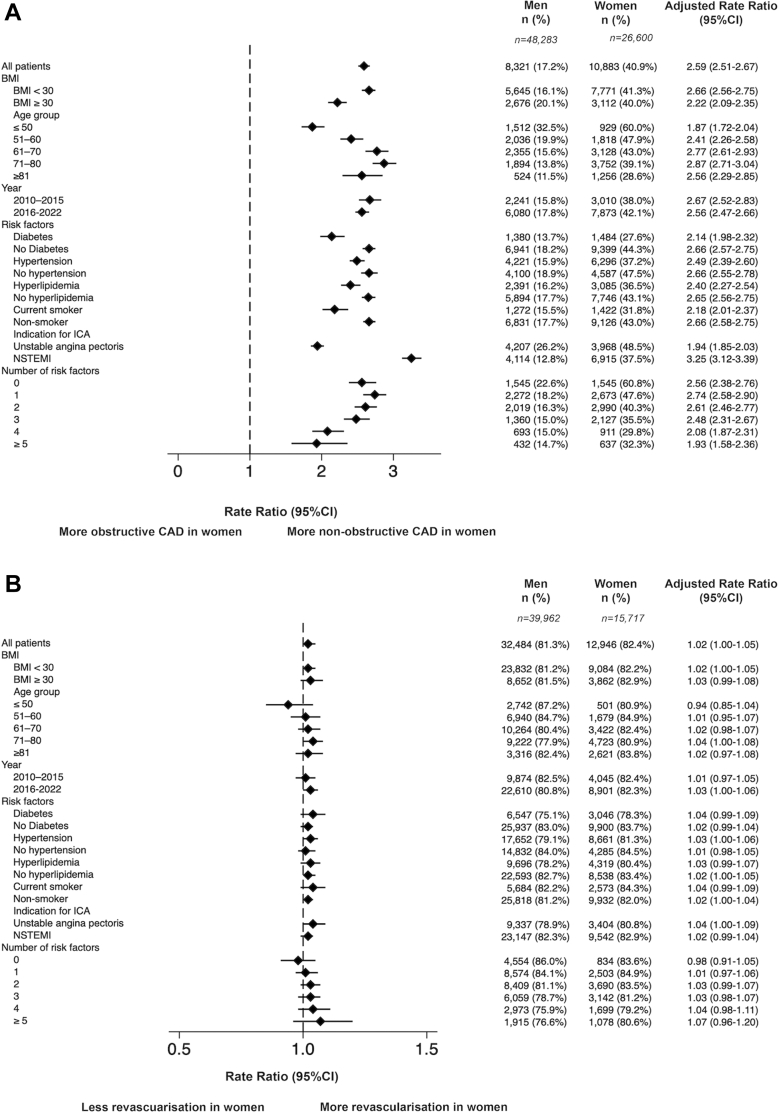
**Sex Differences in Nonobstructive Coronary Artery Disease and Early Revascularization** (A) Absence of obstructive CAD at first-time invasive coronary angiography. Forest plot of adjusted risk ratios for women vs men overall and within prespecified subgroups (BMI, age, calendar year, cardiovascular risk factors, smoking status, indication [NSTEMI or unstable angina], and number of risk factors). (B) 30-day revascularization among patients with obstructive CAD. Adjusted risk ratios for women vs men across the same subgroups. Counts and percentages by sex for each stratum are shown to the right of each panel. Revascularization was defined as PCI or CABG within 30 days of the index ICA. All models were adjusted for age, body mass index, diabetes mellitus, hypertension, hyperlipidemia, chronic kidney disease, congestive heart failure, smoking status, and indication for ICA (NSTEMI or unstable angina). Age is presented in years and body mass index (BMI) in kg/m^2^. CAD = coronary artery disease; ICA = invasive coronary angiography; NTSEMI = non–ST-segment elevation myocardial infarction.

**Figure 4:**
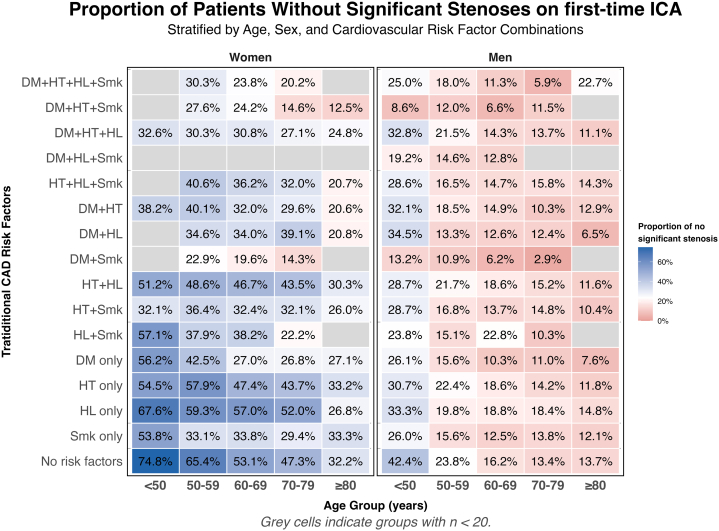
**Proportion of Coronary Angiographies Without Significant Obstructive Coronary Artery Disease** The proportion of no significant CAD on at first-time ICA, presented in a heatmap stratified by combinations of traditional cardiovascular risk factors (rows), decadal age groups (columns), and sex (women, left panel; men, right panel). Each cell represents a distinct patient subgroup, with the value and corresponding color indicating the proportion of patients within that subgroup who had no significant coronary stenosis on their first angiogram. Higher proportions are indicated by blue, and lower proportions by red. Gray cells denote subgroups with a small sample size (n < 20). DM = diabetes mellitus; HL = hyperlipidemia; HT = hypertension; Smk = current smoker; other abbreviations as in Figure 3.

Key sex differences in angiographic findings and revascularization rates are summarized in the Central Illustration. In the Swedish cohort, 19,204 (25.6%) patients had no significant CAD, including 10,883 women (40.9%) and 8,321 men (17.2%); aRR 2.59 (95% CI: 2.51-2.67) (Figure 2). Similar proportions were observed across most risk factor profiles and age groups (Table 1 and Figure 3). Women with no significant CAD were on average 3 years older than women with obstructive CAD, whereas men with obstructive CAD were 5 years older than men with no significant CAD. Among women <50 years, the prevalence of no significant CAD was 74.8% (Figure 4). Women and men with no significant CAD had broadly similar risk profiles, whereas women with obstructive CAD had higher rates of hypertension and chronic kidney disease compared with men with obstructive CAD (Table 1). Furthermore, procedural complication rates were 0.3% (n = 22) in men and 0.5% (n = 52) in women without significant CAD, and 1.0% (n = 403) in men and 1.6% (n = 250) in women with CAD (Supplemental Table 7). The proportion of patients undergoing ICA without significant CAD remained stable over the study period (Supplemental Figure 2).

**Central Illustration:**
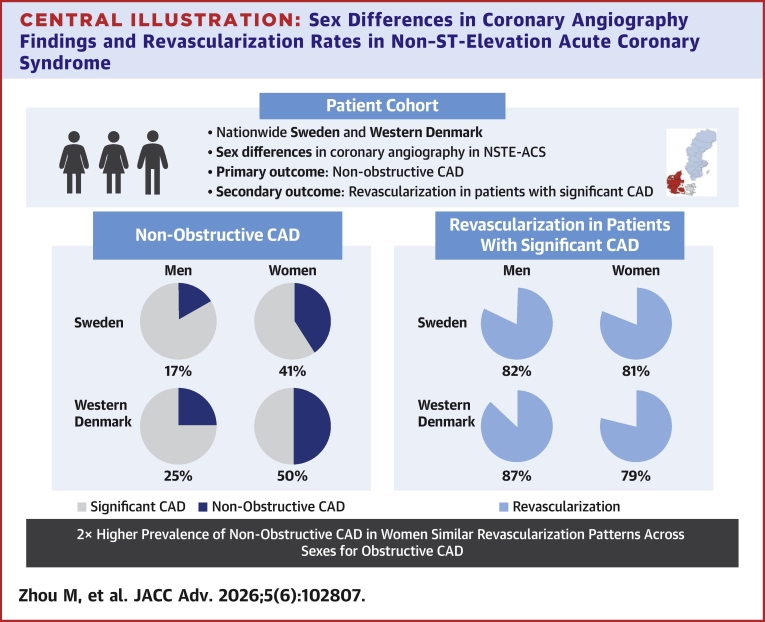
**Sex Differences in Coronary Angiography Findings and Revascularization Rates in Non–ST-Elevation Acute Coronary Syndrome** Among patients without previously known coronary artery disease undergoing first-time coronary angiography for suspected NSTE-ACS in Sweden and Denmark, women had approximately twice the prevalence of nonobstructive coronary artery disease compared with men. Revascularization rates were overall similar between women and men among patients with significant coronary artery disease. CAD = coronary artery disease; NSTE-ACS = non–ST-segment elevation acute coronary syndrome.

In the Danish cohort, 5,214 women (50.1%) had no significant CAD compared with 4,224 men (25.2%); aRR 2.01 (95% CI: 1.95-2.08) (Supplemental Table 3). The overall pattern of sex differences was consistent with the Swedish findings.

### Secondary outcomes – revascularization rates and 1-year MACE rates

Among patients with obstructive CAD, revascularization rates in Sweden were similar between women and men (82.4% vs 81.3%; aRR: 1.02; 95% CI: 1.00-1.05) (Figure 2). In contrast, women in Denmark were less likely to be revascularized compared with men (79.4% vs 86.5%; aRR: 0.80; 95% CI: 0.75-0.86) (Supplemental Table 3).

**Figure 5:**
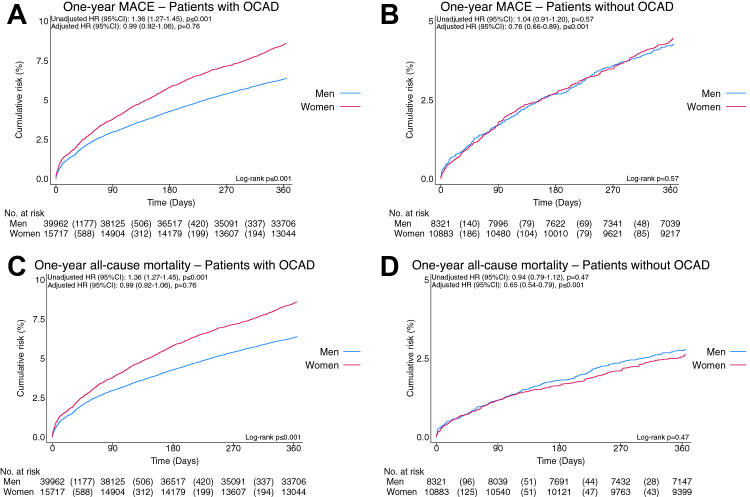
**Secondary outcomes – 1-year Major Adverse Cardiovascular Events and Myocardial Infarction Rates in Men and Women** Kaplan-Meier curves illustrating the 1-year event rate of MACE in patients (A) with and (B) without the presence of significant CAD at index angiography, and all-cause death in patients (C) with and (D) without the presence of significant stenoses at index angiography. MACE was defined as all-cause death, incident myocardial infarction, and stroke. Adjusted models for patients without obstructive CAD were adjusted for year of ICA; age; smoking status; diabetes mellitus; prior stroke; chronic kidney disease; congestive heart failure; chronic obstructive pulmonary disease; peripheral arterial disease; hypertension; hyperlipidemia; obesity; and indication for ICA (NSTEMI or unstable angina). In patients with obstructive CAD, models were additionally adjusted for angiographic findings and revascularization. MACE = major adverse cardiovascular events; OCAD = obstructive coronary artery disease.

In Sweden, the 1-year cumulative incidence percentage of MACE among patients with no significant CAD was 4.5% in women and 4.3% in men (aHR: 0.76; 95% CI: 0.66-0.89). Among patients with obstructive CAD, corresponding event rates were 8.6% in women and 6.4% in men (aHR: 0.99; 95% CI: 0.92-1.06) (Supplemental Table 6, Figure 5). In Denmark, corresponding rates were 5.0% vs 5.6% for nonobstructive CAD (aHR: 0.73; 95% CI: 0.58-0.91), and 13.0% vs 9.3% for obstructive CAD (aHR: 1.05; 95% CI: 0.93-1.19) (Supplemental Figure 1).

For all-cause death, 1-year rates in Sweden were 2.6% in women and 2.8% in men with no significant CAD (aHR: 0.65; 95% CI: 0.54-0.79), and 4.6% vs 3.7% with obstructive CAD (aHR: 0.83; 95% CI: 0.75-0.92) (Supplemental Table 6). In Denmark, mortality was 3.8% vs 4.3% for no significant CAD (aHR: 0.67; 95% CI: 0.52-0.87), and 8.7% vs 6.0% for obstructive CAD (aHR: 0.97; 95% CI: 0.84-1.13) (Supplemental Table 4).

In Sweden, 1-year risks of incident MI were 1.2% in women and 0.8% in men with no significant CAD (aHR: 1.12; 95% CI: 0.81-1.54), and 3.5% vs 2.2% with obstructive CAD (aHR: 1.31; 95% CI: 1.16-1.47). Stroke rates were similar between sexes: 1.0% vs 0.9% for nonobstructive CAD (aHR: 0.95; 95% CI: 0.69-1.32) and 1.5% vs 1.1% for obstructive CAD (aHR: 0.92; 95% CI: 0.77-1.10) (Supplemental Table 6).

### Sensitivity analysis −70% stenosis

In a sensitivity analysis using a stricter definition of obstructive CAD (≥70% stenosis), the prevalence of nonobstructive CAD remained substantially higher in women than in men (44.9% vs 20.6%), consistent with the primary analysis.

## Discussion

In this contemporary study of patients undergoing first-time ICA for NSTE-ACS, 40% of women in Sweden had nonobstructive CAD, approximately twice the rate of that observed in men. Rates were particularly high in younger women without cardiovascular risk factors, where almost 75% under 50 years of age had nonobstructive CAD. Similar findings were observed in Western Denmark, where approximately 50% of women had nonobstructive CAD, also twice the prevalence compared to men. No sex differences were seen in revascularization rates in Sweden, whereas men were more likely to be revascularized in Western Denmark; however, reassuringly, no differences were seen in mortality rates despite this disparity. One-year MACE and all-cause mortality rates were lower in women in both CAD and no-CAD groups after adjustment.

Sex differences with lower rates of obstructive CAD in diagnostic yield have previously been described in both chronic coronary syndrome and ACS cohorts, more recently by Pompei et al^21^ and Sarma et al^22^ in NSTE-ACS populations. However, reported rates of nonobstructive disease in these studies have generally been lower, often reflecting more selected populations or older cohorts. The higher prevalence of nonobstructive CAD in women is well established and likely reflects multifactorial mechanisms, including differences in coronary pathophysiology and presentation. The present study extends these observations by providing contemporary nationwide data from 2 Scandinavian countries, demonstrating consistently high rates of nonobstructive findings among women undergoing first-time ICA for NSTE-ACS. By restricting the analysis to first-time angiography in nationwide registries in the high-sensitivity troponin era, and validating findings across 2 independent health care systems, the study provides robust real-world estimates of angiographic findings in this population. Granular age- and risk factor–stratified analyses further highlight patient groups with particularly high rates of nonobstructive findings. These findings may help inform the development of sex-specific prediction models incorporating preangiographic clinical data, including risk scores, troponin kinetics, and noninvasive imaging, particularly among younger women without traditional cardiovascular risk factors.

Previous studies have suggested that women may be referred for ICA less frequently than men.^22^, ^23^, ^24^ Among patients who do undergo ICA for suspected NSTE-ACS, our findings instead demonstrate a substantially lower diagnostic yield in women, highlighting ongoing challenges in risk stratification among patients referred for ICA. Compared with randomized controlled trials, which have reported lower absolute rates of nonobstructive CAD, the higher prevalence observed in our study likely reflects real-world practice, broader inclusion criteria, and the contemporary use of high-sensitivity cardiac biomarkers.

The proportion of nonobstructive CAD over the last decade has remained relatively stable and has even slightly increased annually in Sweden. This has happened despite a decreasing number of ICAs, and corresponding increase in coronary computed tomography angiography usage.^25^ Despite this overall reduction in ICA volume, which would be expected to enrich the remaining population for higher-risk, higher-yield cases, the proportion of nonobstructive findings have remained stable or increased. A notable increase in nonobstructive rates was observed during and following the COVID-19 pandemic; however, the cause of this remains unclear.

The Swedish cohort was older and demonstrated a higher burden of certain individual comorbidities, whereas the Danish cohort exhibited a greater overall cardiovascular risk factor load, with particularly higher smoking rates. The exact causes of the difference in absolute rates cannot be determined from the available data. Differences in referral thresholds or local diagnostic pathways may also have influenced the population undergoing angiography. Direct comparison with U.S. cohorts is limited by differences in inclusion criteria. In NSTEMI patients from the National Cardiovascular Data Registry (NCDR) Chest Pain–MI registry, patients are on average slightly younger and have a higher prevalence of diabetes, obesity, and smoking.^26^ These differences likely reflect variation in baseline population risk profiles and broader inclusion criteria, and should be considered when interpreting the data. Health care systems also differ, with Scandinavian systems providing universal coverage, whereas the U.S. system is largely insurance based.

Despite lower event rates than in obstructive CAD, the 1-year MACE rate of 4 to 5% among patients with nonobstructive CAD are clinically meaningful and should not be interpreted as evidence of a benign prognosis. Event rates among patients with nonobstructive CAD were broadly similar between women and men in both cohorts, whereas among patients with obstructive CAD, women had higher crude 1-year MACE rates than men, although these differences were largely attenuated after multivariable adjustment. These rates are broadly consistent with those reported in studies of MINOCA, although the present cohort is broader and likely includes patients with heterogeneous underlying mechanisms, some of whom may carry lower individual risk.^27^ The absence of mechanistic phenotyping within the nonobstructive group means that risk stratification beyond traditional cardiovascular risk factors was not possible in this study. Taken together, these findings reinforce that nonobstructive CAD at angiography should not be considered a benign finding and may warrant further evaluation depending on the clinical context. Procedural complication rates were low overall but were slightly higher in women than men, both among patients with and without obstructive CAD.

Revascularization rates among patients with obstructive CAD were similar between women and men in Sweden, whereas women were less likely to be revascularized in Denmark. Despite these differences, adjusted risks of MACE and all-cause mortality were similar between sexes in Denmark, and differences in crude event rates likely reflect baseline differences at presentation.

### Study Limitations

Several limitations should be acknowledged. First, this study is observational, which might introduce selection bias that cannot be accounted for. Second, preangiographic clinical data including clinical symptoms, such as GRACE score, and results of prior noninvasive testing were unavailable. These factors are essential to the decision to proceed with ICA, limits interpretation of the data. Third, the coronary angiograms were reported by the performing physician, and not by a core laboratory, making the data susceptible to intraobserver and interobserver variability. Fourth, no data regarding the specific phenotype of nonobstructive CAD were available. Nonobstructive CAD is notably a mechanistically heterogenous group. SCAD was excluded from the Swedish cohort but not excluded in the Danish cohort due to lacking data on this. Given that SCAD disproportionately affects younger women, this exclusion may have influenced the observed sex differences in nonobstructive CAD rates and limits direct cross-country comparability. Finally, differences in local practice patterns and reporting between countries may have influenced referral decisions and angiographic classifications, which should be considered when interpreting the results.

## Conclusions

In 2 population-based cohorts with suspected NSTE-ACS undergoing first-time ICA, 40 to 50% of women had no significant CAD, approximately twice the proportion compared to men, highlighting a need for more refined patient selection for invasive evaluation. Rates of revascularization and clinical outcomes were comparable between men and women with CAD, suggesting attenuation of previously reported sex disparities.

Perspectives **COMPETENCY IN SYSTEMS-BASED PRACTICE:** Among patients undergoing first-time coronary angiography for suspected NSTE-ACS, women have a substantially higher prevalence of nonobstructive CAD compared with men, despite similar indications for invasive evaluation. Recognition of these sex differences may support more informed diagnostic decision-making, particularly when considering the expected yield of ICA. At a systems level, understanding population-level differences in angiographic yield between women and men may guide quality improvement efforts and support more efficient use of invasive diagnostic resources in acute coronary syndrome pathways.

**TRANSLATIONAL OUTLOOK:** Future studies should evaluate sex-specific diagnostic strategies integrating clinical assessment, biomarkers, and noninvasive imaging to optimize patient selection for coronary angiography.

## Funding support and author disclosures

This work was supported by the Swedish Heart and Lung Foundation, the 10.13039/501100004359Swedish Research Council, Skåne University Hospital funds, ALF funding, the Swedish Society of Medicine, 10.13039/501100003173The Crafoord Foundation, and the 10.13039/501100009708Novo Nordisk Foundation (grant number NNF22OC0074083). The sponsors had no role in the study design; data collection, analysis, or interpretation; manuscript preparation; or the decision to submit the manuscript for publication. The authors have reported that they have no relationships relevant to the contents of this paper to disclose.
