# Supplementary material for: Contemporary Data on Sex Differences in Coronary Angiography Findings: A Dual-Nation Study
Source: JACC Adv. 2026 Jun 17;5(6):102807. doi: 10.1016/j.jacadv.2026.102807 (PMC13309307; doi:10.1016/j.jacadv.2026.102807)
Supplement: Supplementary material [file mmc1.pdf]

Supplementary materials

Elements included in the supplementary materials:

Supplementary Tables

Supplementary Table 1. Definitions and data sources

Supplementary Table 2. Baseline characteristics – Western Denmark Heart Registry

Supplementary Table 3. Revascularization rates – Western Denmark Heart Registry

Supplementary Table 4. MACE and all-cause mortality rates – Western Denmark Heart Registry

Supplementary Table 5. Baseline characteristics stratified by sex – SWEDEHEART

Supplementary Table 6. One-year MACE and MACE Components - SWEDEHEART

Supplementary Table 7. In-laboratory procedural complication rates – SWEDEHEART

Supplementary Figures

Supplementary Figure 1. MACE and all-cause mortality rates in patients with and without CAD – Western Denmark Heart Registry

Supplementary Figure 2. Proportion of non-obstructive coronary angiographies by year – SWEDEHEART

Deleted: Individual endpoints for MACE rates – SWEDEHEART…

Deleted: Supplementary Table 8. Covariates included in multivariable models¶

Supplementary Table 1. Definitions and Data sources

**Outcome**	**Database**	**Definition**
Major adverse cardiovascular event (MACE)	SCAAR	Composite of all-cause mortality, incident myocardial infarction and stroke
All-cause death	National Patient Registry	
Incident myocardial infarction	National Patient Registry	I21, I22
Stroke	National Patient Registry	I60, I61, I62, I63, I64
Significant coronary artery disease	SCAAR	Diagnosed by examining interventional physician. In SCAAR defined as ≥ 50% obstruction of the luminal diameter in any major epicardial vessel
Revascularization	SCAAR	Defined as ad-hoc PCI, or PCI or CABG within 30 days of index angiography
SCAD (Spontaneous coronary artery dissection)	SCAAR	Defined as suspected or diagnosed SCAD by performing interventionist.

Formatted Table

Supplementary Table 2. Baseline characteristics – Western Denmark Heart Registry

	Men		Women	
	**NOCAD**	**OCAD**	**NOCAD**	**OCAD**
**n**	2,932	7,993	3,713	3,225
**Age at procedure, median (IQR)**	62 (52 – 71)	66 (57 – 74)	67 (57 – 75)	71 (61 – 79)
**Inclusion period**				
2013 – 2017	1,515 (51.7%)	4,399 (55.0%)	1,977 (53.2%)	1,791 (55.5%)
2018 – 2022	1,417 (48.3%)	3,594 (45.0%)	1,736 (46.8%)	1,434 (44.5%)
**BMI, kg/m^2; median (Q1-Q3)**	27.0 (25.0 – 31.0)	27.0 (25.0 – 30.0)	26.0 (23.0 – 30.0)	26.0 (23.0 – 30.0)
**BMI**				
Underweight	24 (0.8%)	47 (0.6%)	139 (3.7%)	98 (3.0%)
Normal weight	603 (20.6%)	1,605 (20.1%)	1,303 (35.1%)	1,088 (33.7%)
Overweight	1,257 (42.9%)	3,678 (46.0%)	1,145 (30.8%)	1,089 (33.8%)
Obese	890 (30.4%)	2,218 (27.7%)	880 (23.7%)	769 (23.8%)
Missing	158 (5.4%)	445 (5.6%)	246 (6.6%)	181 (5.6%)
**Diabetes**	453 (15.5%)	1,385 (17.3%)	497 (13.4%)	669 (20.7%)
**Hypertension**	1,470 (50.1%)	4,203 (52.6%)	2,104 (56.7%)	2,073 (64.3%)
**Hyperlipidemia**	719 (24.5%)	1,979 (24.8%)	1,031 (27.8%)	892 (27.7%)
**Previous stroke**	71 (2.4%)	279 (3.5%)	125 (3.4%)	131 (4.1%)
**Congestive heart failure**	231 (7.9%)	1,048 (13.1%)	485 (13.1%)	520 (16.1%)
**Kidney failure**	100 (3.4%)	329 (4.1%)	116 (3.1%)	141 (4.4%)
**Smoking**	719 (24.5%)	2,352 (29.4%)	685 (18.4%)	836 (25.9%)
**Peripheral artery disease**	110 (3.8%)	536 (6.7%)	165 (4.4%)	314 (9.7%)
**Urgency**				
Elective	285 (9.7%)	835 (10.4%)	290 (7.8%)	341 (10.6%)
Acute	2,456 (83.8%)	6,802 (85.1%)	3,224 (86.8%)	2,762 (85.6%)
Subacute	191 (6.5%)	356 (4.5%)	199 (5.4%)	122 (3.8%)
**Indication**				
NSTEMI	1,668 (56.9%)	6,772 (84.7%)	2,431 (65.5%)	2,758 (85.5%)
Unstable angina	1,264 (43.1%)	1,221 (15.3%)	1,282 (34.5%)	467 (14.5%)
**Number of risk factors**				
0	490 (16.7%)	965 (12.1%)	526 (14.2%)	235 (7.3%)
1	834 (28.4%)	2,142 (26.8%)	962 (25.9%)	689 (21.4%)
2	763 (26.0%)	2,169 (27.1%)	1,031 (27.8%)	928 (28.8%)
3	517 (17.6%)	1,519 (19.0%)	696 (18.7%)	742 (23.0%)
4	237 (8.1%)	793 (9.9%)	347 (9.3%)	427 (13.2%)
≥ 5	91 (3.1%)	405 (5.1%)	151 (4.1%)	204 (6.3%)
**Distribution of vessel disease**				
0VD	2,932 (100%)	0 (0.0%)	3,713 (100%)	0 (0.0%)
1VD	0 (0.0%)	4,116 (51.5%)	0 (0.0%)	1,860 (57.7%)
2VD	0 (0.0%)	2,145 (26.8%)	0 (0.0%)	793 (24.6%)

Deleted: ¶ ... [1]

Deleted: BMI, median (IQR) ... [2]

Formatted: Font: Times New Roman, 9 pt

Formatted: Font: 9 pt

Formatted: Font: Times New Roman, 9 pt

Formatted: Font: 9 pt

Formatted: Superscript

Formatted: Font: 9 pt

Formatted: Font: 9 pt

Formatted: Font: 9 pt

Formatted: Font: 9 pt

Formatted: Font: 9 pt

3VD	0 (0.0%)	1,732 (21.7%)	0 (0.0%)	572 (17.7%)

Abbreviations: OCAD, obstructive coronary artery disease; NOCAD, non-obstructive coronary artery disease; Q1-Q3, 25th-75th percentiles; BMI, body mass index; NSTEMI, non-ST elevation myocardial infarction; VD, vessel disease. COPD, chronic obstructive pulmonary disease; ICA, invasive coronary angiography

Definitions: BMI: Underweight, BMI < 18.5 kg/m^2; Normal weight, BMI 18.5 – 24.9 kg/m^2; Overweight, BMI 25.0 – 29.9 kg/m^2; Obese, BMI ≥ 30 kg/m^2. 1VD, one-vessel disease; 2VD, two-vessel disease; 3VD, three-vessel or left main disease.

Deleted: CAD, coronary artery disease

Deleted: IQR, interquartile range

Formatted: Superscript

Formatted: Superscript

Deleted: -

Deleted: -

Formatted: Superscript

Formatted: Superscript

Deleted: -

Supplementary Table 3. Revascularization rates – Western Denmark Heart Registry

**Outcomes**	**Men**	**Women**	**Adjusted rate ratio (95% CI)**
**N**	12,556	5,192	
**PCI/CABG n(%)**	10,862 (86.5)	4,122 (79.4)	0.80 (0.75–0.86)

Abbreviations: PCI, percutaneous coronary intervention; CABG, coronary artery bypass grafting

The multivariable Cox regression model was adjusted for year of ICA; age; smoking status; diabetes mellitus; prior stroke; chronic kidney disease; congestive heart failure; chronic obstructive pulmonary disease; peripheral arterial disease; hypertension; hyperlipidemia; obesity; and indication for ICA (NSTEMI or unstable angina).

Supplementary Table 4. MACE and all-cause mortality rates – Western Denmark Heart Registry

**MACE**	Events	1-year CIP (95% CI)	Unadjusted HR (95% CI)	Adjusted HR (95% CI)
No CAD				
Men	159	5.6% (4.8-6.5)	ref	ref
Women	180	5.0% (4.4-5.8)	0.90 (0.73-1.11)	0.73 (0.58-0.91)
CAD				
Men	718	9.3% (8.6-9.9)	ref	ref
Women	408	13.0% (11.9-14.2)	1.44 (1.27-1.62)	1.05 (0.93-1.19)

**All-cause death**	Events	1-year CIP (95% CI)	Unadjusted HR (95% CI)	Adjusted HR (95% CI)
No CAD				
Men	122	4.3% (3.6-5.1)	ref	ref
Women	134	3.8% (3.2-4.4)	0.87 (0.68-1.11)	0.67 (0.52-0.87)
CAD				
Men	467	6.0% (5.5-6.6)	ref	ref
Women	273	8.7% (7.8-9.7)	1.47 (1.27-1.71)	0.97 (0.84-1.13)

Abbreviations: MACE, major adverse cardiovascular events; CIP, cumulative incidence proportion; HR, hazard ratio; CI, confidence interval; CAD, coronary artery disease.

Multivariable Cox regression models were adjusted for year of ICA; age; smoking status; diabetes mellitus; prior stroke; chronic kidney disease; congestive heart failure; chronic obstructive pulmonary disease; peripheral arterial disease; hypertension; hyperlipidemia; obesity; and indication for ICA (NSTEMI or unstable angina). In patients with obstructive CAD, models were additionally adjusted for angiographic findings and revascularization.

Formatted: Font: Times New Roman

Formatted: Font: Times New Roman

Formatted: Left

Formatted: Left

Deleted: ¶
Men no. (%) ... [3]

Deleted: ¶
12556 ... [4]

Formatted: Font: Not Bold

Formatted Table

Formatted Table

Formatted: Font: Not Bold

Supplementary Table 5. Baseline Characteristics stratified by sex – SWEDEHEART (Sweden)

	Men	Women
**N**	48,283	26,600
Age, years; median (Q1-Q3)	67 (58-74)	71 (63-78)
**BMI category**		
Underweight	211 (0.4%)	539 (2.0%)
Normal	13,173 (27.3%)	9,189 (34.5%)
Overweight	21,611 (44.8%)	9,100 (34.2%)
Obese	13,288 (27.5%)	7,772 (29.2%)
**Diabetes mellitus**	10,095 (20.9%)	5,372 (20.2%)
**Hypertension**	26,536 (55.0%)	16,943 (63.7%)
**Hyperlipidemia**	14,783 (30.6%)	8,455 (31.8%)
**Previous stroke**	2,634 (5.5%)	1,487 (5.6%)
**Congestive Heart Failure**	1,060 (2.2%)	866 (3.3%)
**Chronic Kidney Failure**	5,954 (12.3%)	7,303 (27.5%)
**Peripheral artery disease**	1,472 (3.0%)	847 (3.2%)
**Active smoker**	8,183 (17.5%)	4,475 (17.4%)
**Angiography indication**		
Unstable angina	16,042 (33.2%)	8,179 (30.7%)
NSTEMI	32,241 (66.8%)	18,421 (69.3%)
**Number of CV risk factors**		
0	12,763 (26.4%)	6,084 (22.9%)
1	14,913 (30.9%)	8,820 (33.2%)
2	11,513 (23.8%)	7,140 (26.8%)
3	6,959 (14.4%)	3,751 (14.1%)
4	2,135 (4.4%)	805 (3.0%)
**Angiographic findings**		
Normal/atheromatosis	8,321 (17.2%)	10,883 (40.9%)
1VD	17,277 (35.8%)	8,040 (30.2%)
2VD	10,723 (22.2%)	3,833 (14.4%)
3VD or LM	11,962 (24.8%)	3,844 (14.5%)

Abbreviations: CAD, coronary artery disease; Q1-Q3, 25th-75th percentiles; BMI, body mass index; NSTEMI, non-ST elevation myocardial infarction; VD, vessel disease. COPD, chronic obstructive pulmonary disease; ICA, invasive coronary angiography

Deleted: ¶ ... [5]

Deleted: ¶ ... [6]

Formatted: Left

Formatted: Font: 9 pt

Deleted: IQR, interquartile range

Definitions: BMI: Underweight, BMI < 18.5 kg/m^2; Normal weight, BMI 18.5 – 24.9 kg/m^2; Overweight, BMI 25.0 – 29.9 kg/m^2; Obese, BMI ≥ 30 kg/m^2. 1VD, one-vessel disease; 2VD, two-vessel disease; 3VD or LM, three-vessel disease or left main disease.

Formatted: Superscript

Formatted: Superscript

Deleted: -

Deleted: -

Formatted: Superscript

Formatted: Superscript

Deleted: -

Deleted: D

Supplementary Table 6. One-year MACE and MACE Components - SWEDEHEART

**MACE**	Events	1-year CIP (95% CI)	Unadjusted HR (95% CI)	Adjusted HR (95% CI)
No CAD				
Men	341	4.3% (3.9 – 4.8)	ref	ref
Women	465	4.5% (4.1 – 4.9)	1.04 (0.91 – 1.20)	0.76 (0.66 – 0.88)
CAD				
Men	2,468	6.4% (6.1 – 6.6)	ref	ref
Women	1,308	8.6% (8.2 – 9.0)	1.36 (1.27 – 1.45)	0.99 (0.92 – 1.06)

**All-cause death**	Events	1-year CIP (95% CI)	Unadjusted HR (95% CI)	Adjusted HR (95% CI)
No CAD				
Men	223	2.8% (2.5 – 3.2)	ref	ref
Women	274	2.6% (2.3 – 3.0)	0.94 (0.79 – 1.12)	0.65 (0.54 – 0.79)
CAD				
Men	1,430	3.7% (3.5 – 3.9)	ref	ref
Women	710	4.6% (4.3 – 5.0)	1.27 (1.16 – 1.39)	0.83 (0.75 – 0.92)

**Myocardial infarction**	Events	1-year CIP (95% CI)	Unadjusted HR (95% CI)	Adjusted HR (95% CI)
No CAD				
Men	63	0.8% (0.6 – 1.0)	ref	ref
Women	123	1.2% (1.0 – 1.4)	1.49 (1.10 – 2.02)	1.12 (0.81 – 1.54)
CAD				
Men	830	2.2% (2.1 – 2.4)	ref	ref
Women	512	3.5% (3.2 – 3.8)	1.58 (1.42 – 1.77)	1.31 (1.16 – 1.47)

**Stroke**	Events	1-year CIP (95% CI)	Unadjusted HR (95% CI)	Adjusted HR (95% CI)
No CAD				
Men	67	0.9% (0.7 – 1.1)	ref	ref
Women	103	1.0% (0.8 – 1.2)	1.17 (0.86 – 1.59)	0.95 (0.69 – 1.32)
CAD				
Men	428	1.1% (1.0 – 1.3)	ref	ref
Women	213	1.5% (1.3 – 1.7)	1.27 (1.08 – 1.50)	0.93 (0.78 – 1.10)

Abbreviations: CAD, coronary artery disease; CIP, cumulative incidence proportion; HR, hazard ratio; CI, confidence interval;

Multivariable Cox regression models were adjusted for year of ICA; age; smoking status; diabetes mellitus; prior stroke; chronic kidney disease; congestive heart failure; chronic obstructive pulmonary disease; peripheral arterial disease; hypertension; hyperlipidemia; obesity; and indication for ICA (NSTEMI or unstable angina). In patients with obstructive CAD, models were additionally adjusted for angiographic findings and revascularization.

Supplementary Table 7. In-laboratory procedural complications

	Events	Proportion (%)
No CAD		
Men	22	0.3%
Women	52	0.5%
CAD		
Men	403	1.0%
Women	250	1.6%

In-laboratory procedural complications occurring during index invasive coronary angiography (ICA) and prospectively recorded in the SWEDEHEART registry. Complications included non-cardiac vascular complications, coronary dissection or perforation, arrhythmic events requiring treatment, and hemodynamic complications, as recorded by the operator during the procedure.

Abbreviations: CAD, coronary artery disease

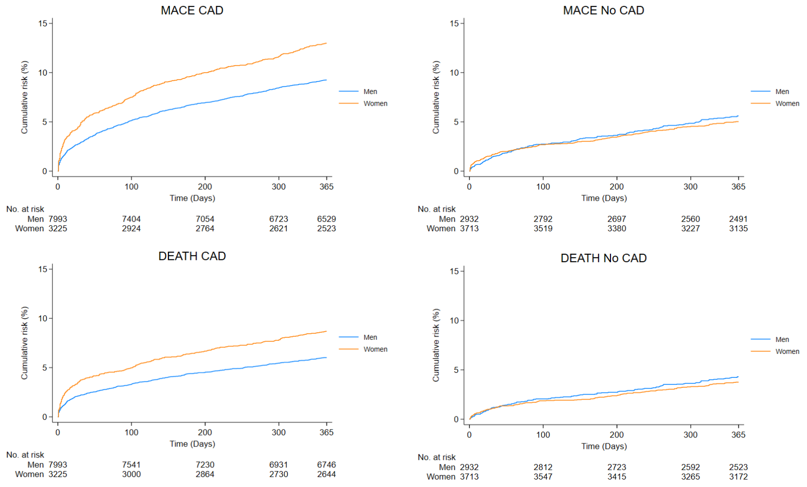

Supplementary Figure 1. MACE and all-cause mortality rates in patients with and without CAD – Western Denmark Heart Registry

Abbreviations: MACE, major adverse cardiovascular events; CAD, coronary artery disease

Deleted: Supplementary Table 8. Covariates included in multivariable models

Analysis ... [7]

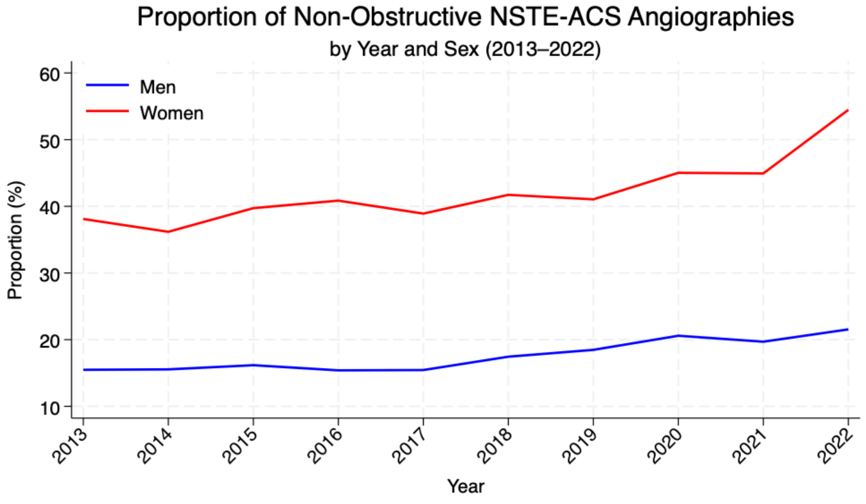

Supplementary Figure 2. Proportion of non-obstructive coronary angiographies by year –

SWEDEHEART

Abbreviations: NSTE-ACS, Non-ST-elevation acute coronary syndrome

Page 3: [1] Deleted	Mikael Zhou	4/7/26 1:38:00 PM
Page 3: [2] Deleted	Mikael Zhou	4/7/26 1:38:00 PM
Page 5: [3] Deleted	Mikael Zhou	4/7/26 10:44:00 AM
Page 5: [4] Deleted	Mikael Zhou	4/7/26 10:40:00 AM
Page 6: [5] Deleted	Mikael Zhou	4/7/26 10:35:00 AM
Page 6: [6] Deleted	Mikael Zhou	4/7/26 10:35:00 AM
Page 10: [7] Deleted	Mikael Zhou	4/7/26 1:19:00 PM
